# Forecasting inpatient glycemic control: extension of damped trend methods to subpopulations

**DOI:** 10.2144/fsoa-2020-0096

**Published:** 2020-11-02

**Authors:** George E Saulnier, Janna C Castro, Curtiss B Cook

**Affiliations:** 1 Department of Information Technology, Mayo Clinic Hospital, Phoenix, AZ 85054, USA; 2 Mayo Clinic, Division of Endocrinology, Scottsdale, AZ 85259, USA

**Keywords:** damped trend analysis, forecasting, hospital, hyperglycemia, inpatient

## Abstract

**Aim:**

Evaluate forecasting models applied to smaller geographic locations within the hospital.

**Materials & methods:**

Damped trend models were applied to blood glucose measurements of progressively smaller inpatient geographic subpopulations. Mean absolute percentage error (MAPE) and 95% prediction intervals (PIs) assessed validity of the models to forecasts 48 weeks into the future.

**Results:**

MAPE values increased, and 95% PIs widened, when data from progressively smaller geographic areas were analyzed. MAPE values were highest and 95% PIs were broadest with the smallest geographic areas. In contrast, observations missed at larger geographical locations were more evident with smaller subpopulations.

**Conclusion:**

The utility of damped trend models to forecast inpatient glucose control diminished when applied to smaller geographic areas within the hospital.

## Lay abstract

Hyperglycemia (high blood sugar) is associated with worse outcomes in hospitalized patients. Predicting future glucose control could identify problems sooner and lead to earlier quality improvement changes. This study examined whether a forecasting tool used across an entire hospital might be useful in smaller geographic areas of the facility. The models became less reliable when tested with progressively smaller geographic subpopulations.

Multiple studies have confirmed an association between inpatient hyperglycemia and adverse outcomes, including greater risks of wound infections, longer lengths of hospital stay and higher mortality rates in both critically ill and noncritically ill patients [1–4]. Current guidelines suggest that a blood glucose value of 140–180 mg/dl represents an inpatient target range that best balances the risks of hyperglycemia against those conferred by hypoglycemia. Similar recommendations apply to critically and noncritically ill patients in the hospital [1–4].

The importance of inpatient glucose management has driven extensive discussion about methods to analyze and report the quality of hospital glycemic control [5]. The current approaches to glucometric reporting typically involve assessment of measures of central tendency (e.g., means) or trends (e.g., control charts) and are limited in that they only inform a hospital of their glycemic history and not necessarily the future state of control [6,7]. Alternatively, glucometric data could be used to project a hospital’s glycemic control into the future. The ability to forecast inpatient glycemic control at the population level could provide an opportunity to anticipate unfavorable changes (i.e., a drift toward glycemic control outside the accepted bounds) before they become a problem, so that interventions could be introduced earlier.

Damped trend exponential smoothing statistical models derived from operational research represent one approach to forecasting inpatient glycemic control. These models forecast trends derived from time series data and have been widely employed in commerce [8–12]. We have previously documented the feasibility of using damped trend analytic methods to forecast inpatient glycemic control by using point-of-care blood glucose (POC-BG) data. Those studies showed that forecasting results are not affected by measurement error inherent in the glucometers used to assess glucose values [13,14].

The emphasis of these forecasting analyses in the previous papers was on summarizing POC-BG patient-day-weighted means on the whole-facility level [13,14]. The emphasis was on all inpatient glucose values from noncritically ill patients across the entire hospital and did not consider geographic subdivisions within the hospital. Although the whole-facility level perspective was insightful from a patient population standpoint, smaller geographic areas within the hospital might be interested in their own glycemic forecasts. However, smaller sample sizes might be expected to affect the value of the forecasts owing to less data availability. As the focus of the model narrows, a trade-off most likely exists between the increased insight provided at smaller geographic sites and the decreased availability of data associated with the progressively smaller units of analysis. The purpose of this analysis was to validate the integrity of the damped trend model as applied to ever-smaller hospital geographic areas and to test whether this more granular-level analysis would continue to provide valuable information on future glycemic control for these smaller areas.

## Materials & methods

### Description of the facility

**Figure 1.**
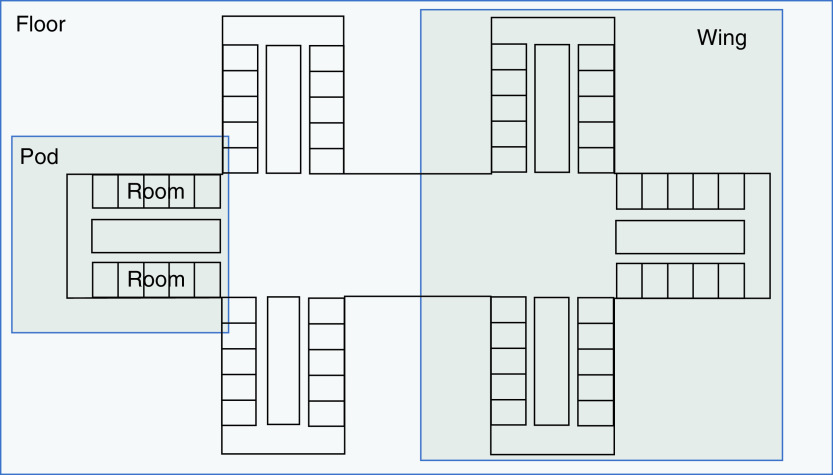
Hospital floor plan. Each hospital floor has two wings: east and west. Image shows layout of a standard wing, which is composed of three adjacent pods.

The study hospital is a 280-bed adult acute care academic teaching facility in a US southwestern metropolitan area. The hospital does not provide inpatient care for obstetric or pediatric patients. The hospital geographic locations considered were the full hospital, individual hospital floors, wings of each floor (the hospital is divided into east and west towers), and pods of each wing. Each wing typically includes 3 pods, with 12 single-bed rooms per pod (Figure 1).

### Data extraction

The same POC-BG data from noncritically ill patients that were used previously were used for this analysis [13,14]. Patient-day-weighted means were calculated for each strata of geographic division. In this instance, the same dataset used for hospital-level forecasting was divided successively into smaller subsets corresponding to the various medical-surgical floors, first at the level of the entire hospital, then a whole floor, followed by a single wing and then an individual pod. Representative quarterly data were used from three separate years: quarter 4 from 2008, quarter 1 from 2015 and quarter 3 from 2017. As with previous reports, this analysis did not involve any patient identifiers and was part of overall quality improvement/quality assurance efforts on inpatient glycemic control and was exempted from formal institutional review board review.

### Data analysis

The damped trend method with corresponding variables previously described to forecast patient-day-weighted means at the hospital level were used to conduct the analyses for the progressively smaller geographic areas [13,14]. At each level, optimal smoothing, trending and damping constants over a 60–63 week learning cycle were computed to determine the most accurate damped trend forecast for a 48-week period into the future. As previously described, the mean absolute percentage error (MAPE) was used to assess the accuracy of the damped trend forecasts [9]. In brief, MAPE is the average of the absolute percentage errors of forecast, with error defined as difference between the observed and forecasted value. Smaller MAPE values are regarded as representing better forecasts [15]. Models resulting in MAPE values ranging from 10 to 15% are considered to be at the upper limits of reasonable data. To evaluate the effects of forecasting on progressively smaller data subsets, we sought to compare the availability of data, the variability of the data and the nature of both the forecasts and the 95% prediction intervals (PIs), as compared with these same factors at the broader hospital level.

## Results

### Hospital-level forecasts

**Figure 2.**
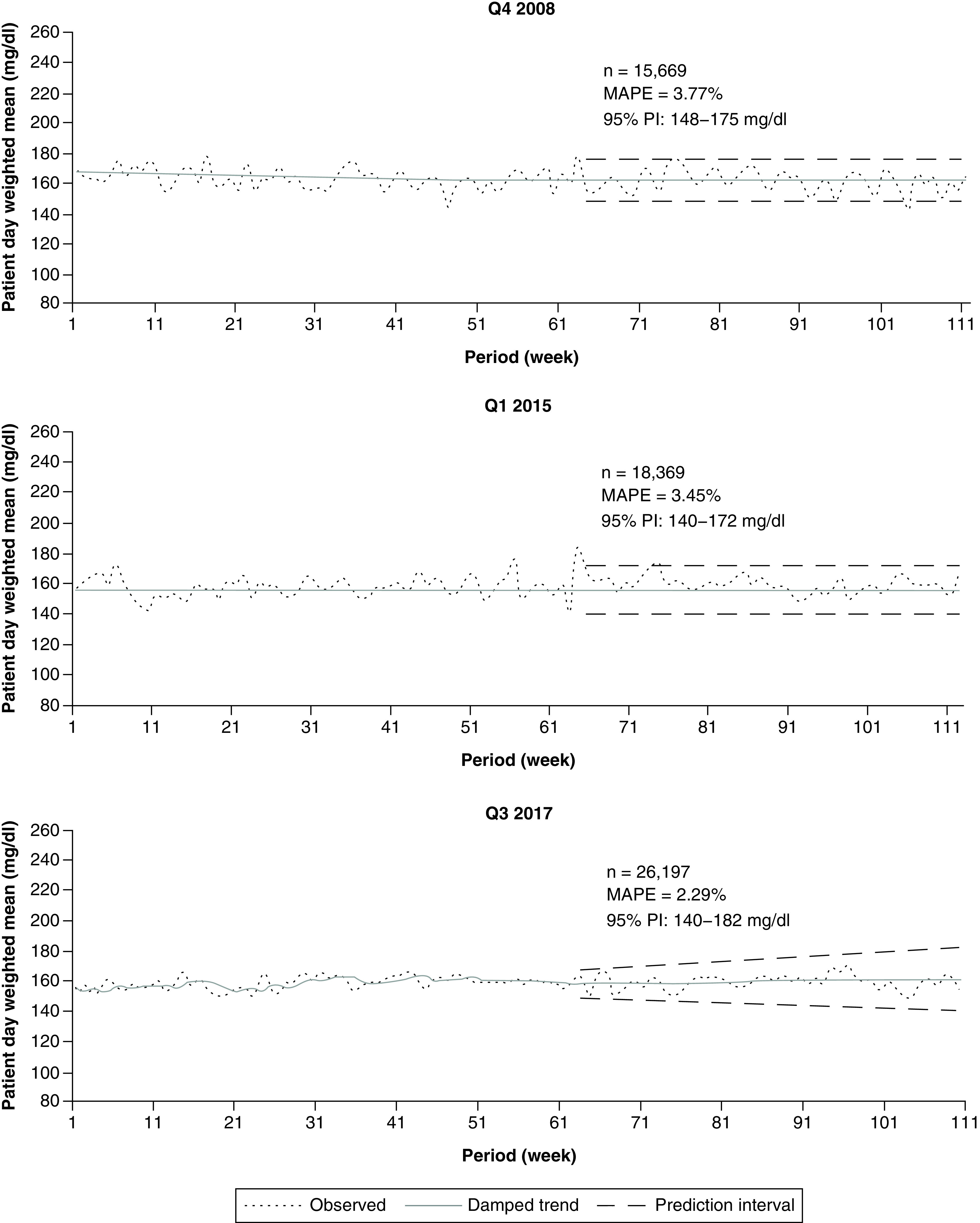
Inpatient glucose forecast: full hospital data. Figure shows 48 week forecasts of glucose data obtained from the entire hospital for years 2008, 2015 and 2017. MAPE: Mean absolute percentage error; Q: Quarter sampled.

We first analyzed data at the full hospital level during the three forecasting periods, one quarter from three different years (Figure 2). With few exceptions, for each time period analyzed, both the observed (actual) patient-day-weighted mean POC-BG values and the damped trend forecasted line were within tightly spaced 95% PIs. All three time periods showed reasonable MAPE values of 3.77% or less. Moreover, patient-day-weighted mean glucose value was forecasted to stay within the recommended 140–180 mg/dl target, which suggests that overall inpatient glucose control at the hospital level was expected to meet recommendations.

### Hospital floor-level forecasts

**Figure 3.**
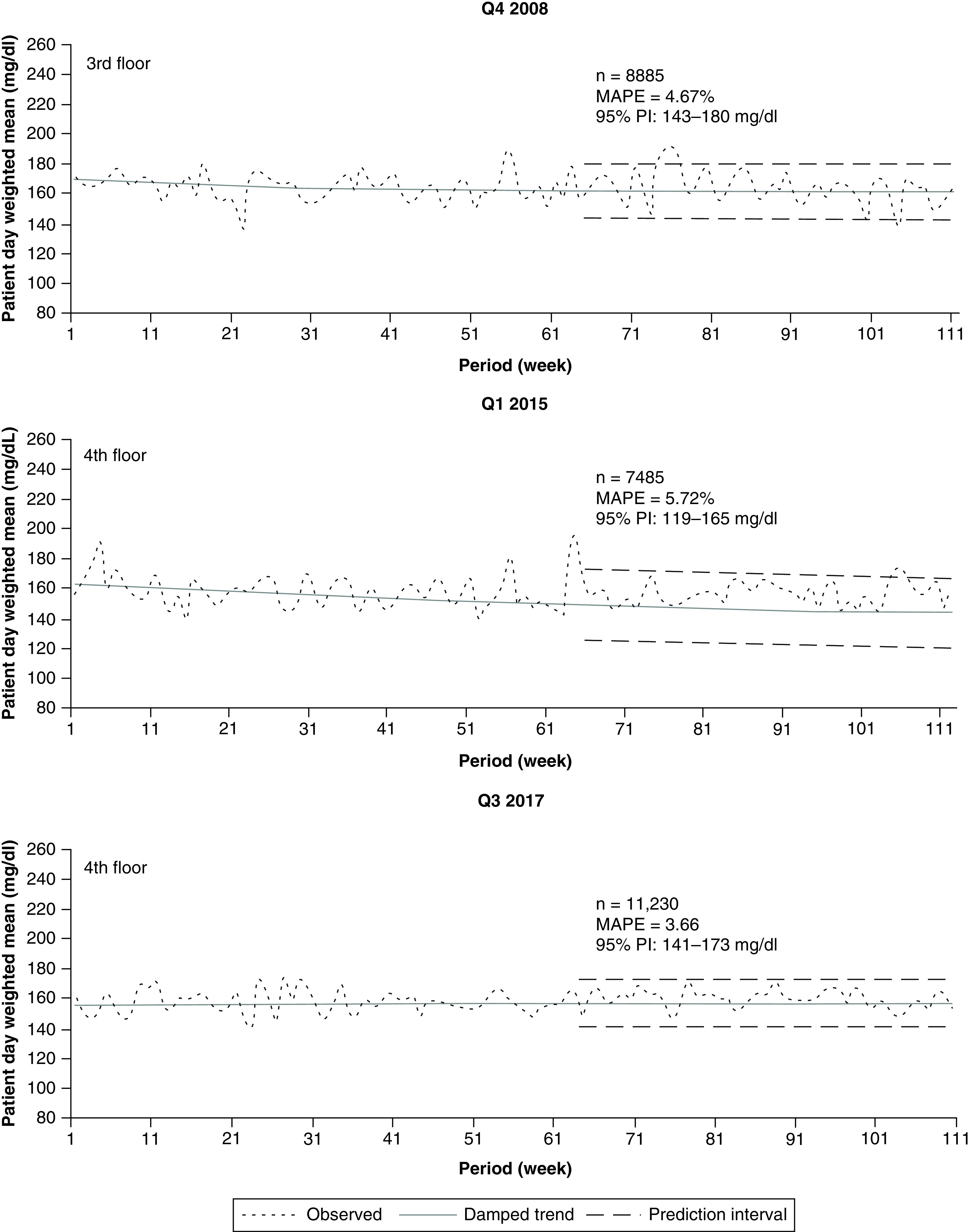
Inpatient glucose forecast: floor-level data. Figure shows examples of 48 week forecasts of glucose data from individual hospital floors for years 2008, 2015 and 2017. MAPE: Mean absolute percentage error; Q: Quarter sampled.

We next analyzed data from the three time periods, but the patient-day-weighted means were restricted to individual floors. For the 2008 data limited to the 3rd floor (Figure 3), the damped trend still closely followed the observations beyond period 63 where the forecast commences. However, several observed values between periods 75 and 103 extended beyond the 95% PIs. The PIs broadened slightly compared with the full hospital data (Figure 2), from 143 to 180 mg/dl and the MAPE was higher (4.67 vs 3.77%).

For the 2015 data limited to the 4th floor (Figure 3), the variability of the observations and the MAPE increased (5.72 vs 3.45%), and the PI widened slightly beyond that for the full hospital (Figure 2). For the 2017 data limited to the 4th floor (Figure 3), the variability of the observations also increased and the MAPE was higher (3.66 vs 2.29%). The PI was no longer thin and funnel-like, as seen in Figure 2, but was consistently wide throughout the forecast time frame. However, predicted values remained within the 140–180 mg/dl desired target range.

### Hospital wing-level analysis

**Figure 4.**
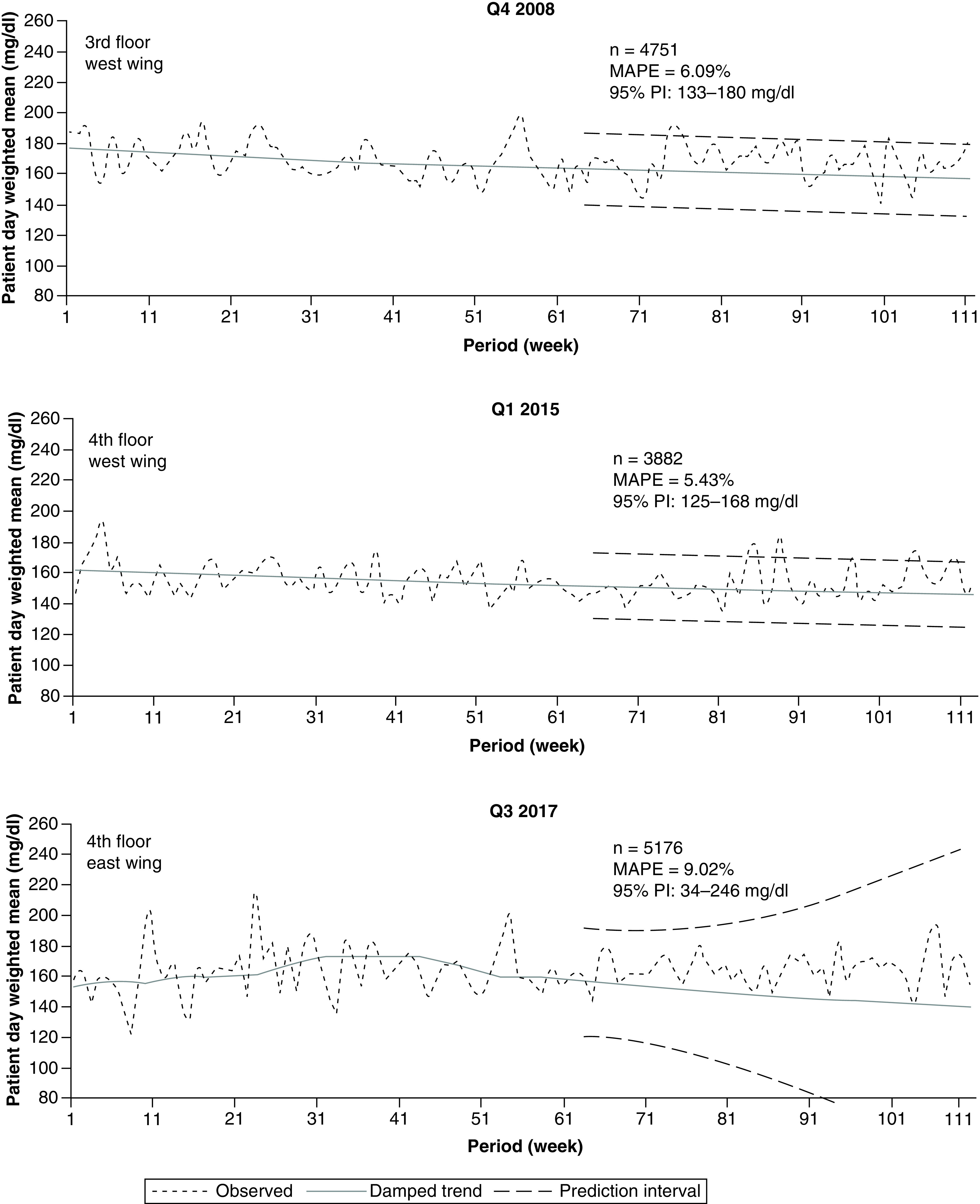
Inpatient glucose forecast: wing-level data. Figure shows examples of 48 week forecasts of glucose data for different hospital wings for years 2008, 2015 and 2017. MAPE: Mean absolute percentage error; Q: Quarter sampled.

We next limited the analyses to one wing (east or west) of each floor previously analyzed (Figure 4). In these smaller geographic units, increasing numbers of values were observed outside of the desired 140–180 mg/dl target glucose range, and forecasts began to be more inconsistent. For the 2008 data limited to the west wing of the 3rd floor (‘3 West’, Figure 4), the MAPE increased from 4.67 to 6.09%, and the 95% PI widened. For the 2015 data limited to the west wing of the 4th floor (‘4 West’, Figure 4), the variability (MAPE and 95% PI) was close to that found in the corresponding floor-level analyses (Figure 3). For the 2017 data limited to the east wing of the 4th floor (‘4 East’, Figure 4), however, a larger MAPE and much greater divergence of the 95% PI was detected. At this level of analysis, focusing on a smaller subpopulation appeared to increase observed values outside desired targets and may have produced much larger variability of results.

### Pod-level analysis

**Figure 5.**
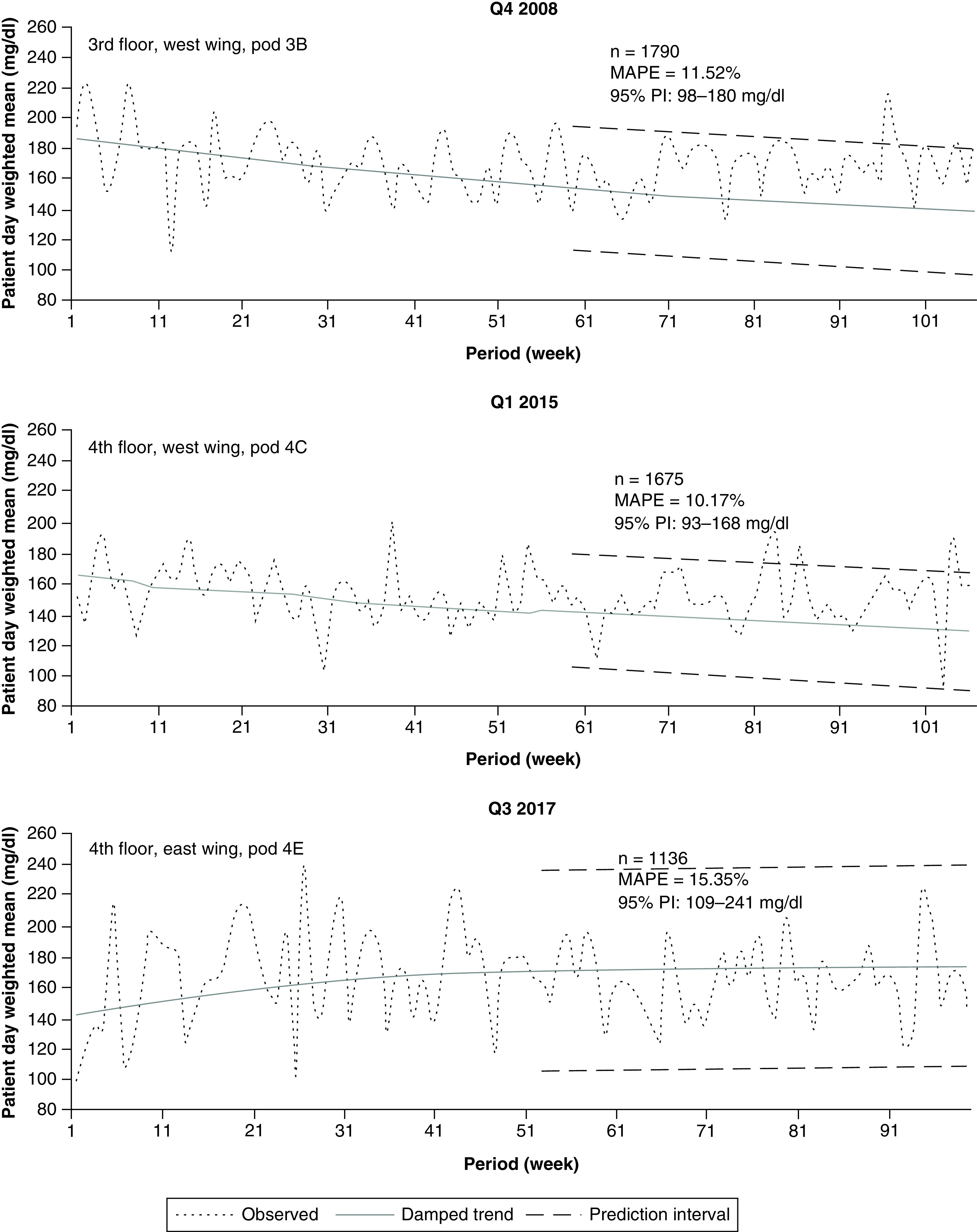
Inpatient glucose forecast: pod-level data. Figure shows examples of 48 week forecasts of glucose data for individual pods for years 2008, 2015 and 2017. MAPE: Mean absolute percentage error; Q: Quarter sampled.

Lastly, we restricted the data to various pods on the 3rd- and 4th-floor wings (Figure 5). The variability of observed and forecasted values visibly increased for all analyses. The decreasing direction of the forecast and equally widening 95% PI for Pod 3B (2008, 3 West, Figure 5) failed to accurately track the observed data beyond period 69. The observations leveled out and stabilized, but the forecast and PIs continued in a decreasing direction. The comparatively wide 95% PI in Figure 5 compared with Figure 4 reflects the variability of the data in Pod 3B early in the learning cycle (periods 1 through 16) relative to the smaller variability during that same 16-week time frame at the wing level. Instead of an expanding 95% PI observed on 4 East (2017 data, Figure 4), the PI for Pod 4E of that wing was uniformly wide (Figure 5).

The MAPEs at the pod level almost doubled in comparison with those observed on the various wings. Pod 4E (2017 data) showed a MAPE of 15.35%, which is no longer considered a reasonable representation of the underlying data. Moreover, the data became more sparse. Instead of the typical 111 periods observed (63-week learning cycle + 48-week forecast) at the hospital/floor/wing level, 106 periods were observed for the 2008 and 2015 pod data (Figure 5), and only 99 periods were observed for 2017 pod data (Figure 5) because of small patient populations in certain pods. The damped trend model results are compared among the different hospital subpopulations for each year in the Table 1.

**Table 1.** Damped trend model results for selected hospital geographic subpopulations.

Level of analysis	Sample size (n)	MAPE (%)	95% PI (mg/dl)
Q4 2008			
Hospital	15,669	3.77	148–175
3rd Floor	8885	4.67	143–180
3 West	4751	6.09	133–180
Pod 3B	1790	11.52	98–180
Q1 2015			
Hospital	18,369	3.45	140–172
4th Floor	7485	5.72	119–165
4 West	3882	5.43	125–168
Pod 4C	1675	10.17	93–168
Q3 2017			
Hospital	26,197	2.29	140–182
4th Floor	11,230	3.66	141–173
4 East	5176	9.02	34–246
Pod 4E	1136	15.35	109–241

MAPE: Mean absolute percentage error; PI: Prediction interval; Q: Quarter.

## Discussion

In previous research, we demonstrated the value of a damped trend model in forecasting population-based glucose measures [13,14]. At a facility level, although these forecasts may assist in anticipating unfavorable changes, the true value of the model may be hidden by the abundance of data. At such a large unit of analysis, the possibility of masking trends exists because underlying fluctuations have been lost in the large sample size. Evaluating ever-smaller geographic subpopulations may uncover both trends worth addressing and unexpected results that can generate hypotheses worthy of investigation.

In contrast, the limitation of evaluating smaller geographic subpopulations is that data will become increasingly sparse. The sparseness and inconsistent availability of data from fewer beds could produce less-accurate forecasts. Our results confirm that, in general, smaller data populations have increased variability, with larger MAPE values and widening of PIs in the damped trend models. Wider PIs may not be as effective as narrower ones in modeling the future direction of inpatient hyperglycemia. Determining the smallest geographic unit of analysis that gives the best insight into future trends while preserving accuracy was the objective of this study.

At least in the scenarios reviewed here, the smallest useful level of analysis was observed between the floor- to wing-level geographic locations. For instance, analyses of wings 3 West and 4 West resulted in visibly similar forecasts as those on the 3rd and 4th floors overall, despite smaller populations. Analysis of wing 4 East, however, despite its larger sample size compared with the other wings, demonstrated evidence of a higher MAPE and a diverging 95% PI. Although 4 East is on the same floor as 4 West, the analysis suggests that something different may be occurring in 4 East that warrants further investigation. These differences could be based on variations in glucose-control processes. Different subpopulations of patient types (e.g., patients with cancer vs those with cardiovascular disease) are accommodated in different hospital areas, which could account for differences. Nonetheless, if inpatient glucose management was consistent, similar variability and forecasts would be expected regardless of both geographic location within the hospital and underlying diagnosis.

Analyses at the pod level yielded MAPE values that were either close to the limit or exceeded the limit of what would be considered reasonable for forecasting. These were associated with typically less than 2000 observations. MAPE values extending beyond the reasonable limit of 15% suggest that the model’s ability to forecast is weakened. This is partly due to the comparatively small populations from which data could be derived. These small populations led to a general widening of the PIs.

Some limitations of this analysis must be noted. First, the data are from a single institution. Therefore, the generalizability of the damped trend approach to forecasting inpatient glycemic control needs to be tested with data derived from other hospitals. Second, because this represents an ecologic (population-based) analysis, conclusions regarding care at the patient level cannot be made. The advantage of a population based analysis is that it could identify geographic areas in need of further study. For instance, if a forecast predicted worsening glycemic control in a specific area within the hospital, additional investigation could be undertaken to determine if the anticipated changes were due to factors occurring at the patient level (e.g., the presence of higher acuity patients) or at the process level (e.g., ineffective use of insulin therapy to control hyperglycemia).

## Conclusion

Although facility-level damped trend analysis of POC-BG data provides an opportunity to anticipate unfavorable changes before they become a problem, the same insight is applicable to smaller hospital subpopulations based on geographic location. As populations decrease in size, however, the risk of sparse data increases, which may affect model results. We tested the integrity of the damped trend model as applied to ever-smaller data sets and validated its usefulness and applicability. In these examples, analyses at the level of the hospital wing most likely represent the smallest unit that balances model validity with identifying potential geographic variations in forecasts.

## Future perspective

Although we have demonstrated the feasibility of using damped trend analysis in forecasting inpatient glycemic control, and now have some idea about the minimum size of a geographic analysis, additional work is required. Whereas this study examined geographic subpopulations, also of interest would be to conduct forecasts in patient subpopulations. For example, it would be interesting to understand how damped trend methods work for forecasting glycemic control in patients with a common diagnosis, such as heart failure or those undergoing solid organ transplants. It would also be of interest to determine whether damped trend methodologies could successfully forecast inpatient hypoglycemia.

A second area in need of future investigation is how to operationalize forecasting methods to improve inpatient glycemic control. Ways that forecasting could be incorporated into more traditional metrics used in quality improvement are unknown. Finally, studies should investigate whether forecasting can change provider behavior or processes affecting inpatient hyperglycemia.

**Summary points**

- Damped trend forecasting models were applied to glucose measurements of progressively smaller geographic hospital inpatient subpopulations.
- Mean absolute percentage error (MAPE) and 95% prediction intervals (PIs) assessed the validity of the models to forecasts extending 48 weeks into the future.
- MAPE values increased, and 95% PIs widened, when data from progressively smaller geographic areas were analyzed.
- MAPE values were highest and 95% PIs were broadest with the smallest geographic area tested.
- Analyses below the hospital wing level yielded the least accurate forecasts.
- Whole hospital-level damped trend analysis of glucose data provides an opportunity to anticipate unfavorable changes before they become a problem.
- The same insight is applicable to hospital subpopulations based on geographic location.
- As populations decrease in size, however, the risk of sparse data increases, with potential impact on model results.
